# Factor XII (Hageman Factor) Deficiency: a rare harbinger of life threatening complications

**DOI:** 10.11604/pamj.2019.33.39.18117

**Published:** 2019-05-21

**Authors:** Liaqat Ali Chaudhry, Wael Yasin Mohamed El-Sadek, Ghazala Aslam Chaudhry, Feddha Eid Al-Atawi

**Affiliations:** 1 Department of Internal Medicine, King Salman Armed Forces Hospital Tabuk, Saudi Arabia; 2 Department of Hematology, King Salman Armed Forces Hospital Tabuk, Saudi Arabia; 3 Department of Family Medicine, King Salman Armed Forces Hospital Tabuk, Saudi Arabia; 4 Department of Internal Medicine King Salman Armed Forces Hospital Tabuk, Saudi Arabia

**Keywords:** Hageman Factor (Factor-XII) deficiency, aPTT-activated partial thromboplastin time, pulmonary embolism

## Abstract

Hageman factor (factor XII) has a key role in activation of intrinsic coagulation system gauged by activated partial thromboplastin time (aPPT). Hageman factor deficiency is more often an autosomal recessive condition, but an autosomal dominant inheritance is also reported. This condition in its own is not known to cause bleeding complications rather is associated with paradoxical fatal thromboembolic complications. Exact prevalence of this condition is not known, as under normal conditions they are asymptomatic. In literature, a prevalence of 2.3% has been reported in one study on 300 patients presenting with complications. Homozygous patients has non-detectable levels of factor XII, while heterozygous individuals has variable levels ranging from 20-60%. Hageman factor is a pro-coagulation protein initiating intrinsic pathway. Intrinsic pathway is activated either by direct contact with a negative charged surface or by proteolytic activation on the endothelial cells via prekallikerin/kallikerin system. Factor XII as an integral part of this system leads to factor XI activation resulting in production of thrombin orchestrated by intrinsic system. In addition, there is concomitant activation of complement components C3 and C5 via C1-estrase activation. Patients with this condition are known to have spontaneous thromboembolic complications although less common but are prone to life threatening complications under provocating circumstances. The aim of this case report is to study the relation of factor XII deficiency and isolated raised activated partial thromboplastin time (aPPT) and how it can be prevented. We are presenting a Saudi female patient, 29 years of age who presented to accident and emergency room (A&E room) of our hospital with sudden severe breathlessness and chest pain.

## Introduction

Hageman factor (factor XII) deficiency is a congenital condition inherited in large majority as autosomal recessive condition. It belongs to the vast group of kinins [1, 2]. Factor XII is important in initiating activation of intrinsic coagulation pathway. Surprisingly it is very rarely if at all associated with bleeding diathesis that too is very mild like epistaxis or skin abrasions. But contrary and confusingly factor XII deficiency is more often associated with thromboembolic complications which are sometimes life threatening [3, 4]. The condition is often incidentally discovered during coagulation screening having isolated prolonged activated partial thromboplastin time (aPTT) or during an unexplained coagulopathy. Association of arterial and venous thromboembolic events has been debated in the literature leading to myocardial infarction and life-threatening pulmonary embolism [4, 5]. A high index of suspicion is kept in individuals, known to have factor XII deficiency, presenting with thromboembolic events spontaneously or more so under provocation as in the subject patient. Early diagnosis based on unexplained isolated prolonged PTT and prompt intervention with anticoagulation is lifesaving in acute myocardial infarction or massive or sub-massive pulmonary embolism [6].

## Patient and observation

A Saudi female age 29, presented to accident and emergency room (A&E) of our hospital with sudden severe breathlessness and chest pain. She has been ambulant and asymptomatic, discharged recently from our hospital three weeks ago after an uneventful caesarean section delivery being primigravida.

**History:** she was in obvious respiratory distress, tachypneic and tachycardiac having desaturation at room air under resting conditions. Admitting diagnosis was pulmonary embolism until proved otherwise. She has been ambulant and had no past history of significance being non-smoker, no history of taking any medications. She denied family history of any blood disorders.

**General physical examination:** patient looking anxious and in distress, BP = 105/60 mmgh, HR = 115/min, RR = 29/min, O2 saturation at room air at rest = 85%, Temperature = 36.7C, Weight= 58.7 Kg. No clinical signs of deep vein thrombosis.

**Systemic examination:** her systemic examination was unremarkable.

### Investigations

**Figure 1:**
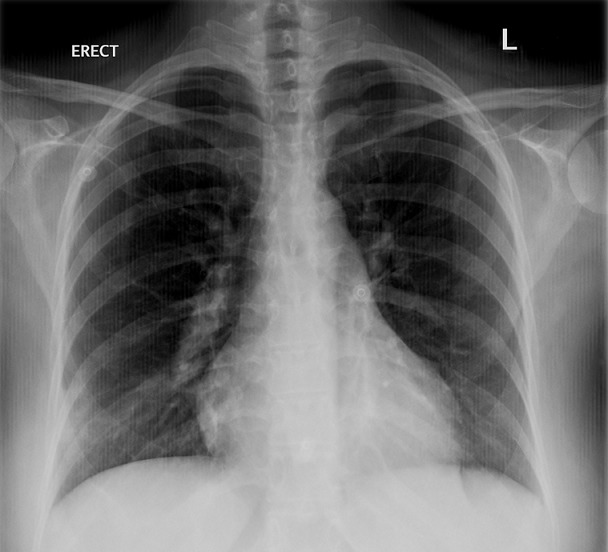
Chest X-ray PA view was reported normal

**Figure 2:**
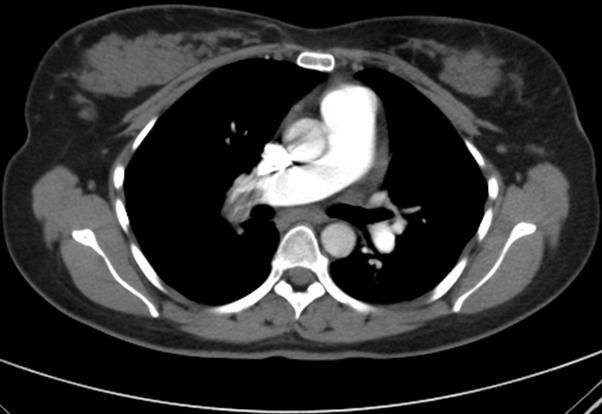
CT-Scan chest with contrast showing bilateral sub-massive pulmonary emboli

Chest X-ray normal (Figure 1), ECG-Tachycardia & S1Q3T3 typical of pulmonary embolism, raised cardiac enzyme troponin = 0.18, urgent echocardiography reported indicating right ventricle dilatation with inter-ventricular septal deviation and elevated pulmonary artery pressure (PAP = 45mmhg) suggestive of significant large pulmonary embolism. CT-Scan chest with intravenous contrast reported bilateral sub-massive pulmonary emboli (Figure 2). Doppler studies on lower limb vessels reported normal. Arterial blood gases on arrival at room air, PH = 7.54, PaCO2 = 27, PaO2=52, SBE = -4, HCO3 = 19, O2 Saturation = 86%, baseline coagulation studies, showed isolated prolonged activated partial thromboplastin time (aPTT) = 41 (normal = 16-36), prothrombin time (PT) = 16, international normalized ratio(INR) = 1.20, D-Diamer = 2.3, WBC = 4.5, RBC = 4.2, Hb = 11.4, Platelets = 218, MCV = 80, MCH = 30, BUN = 4.8, Scr = 66, CRP = 1.4, liver functions and serum electrolytes were normal. Mixing studies showed correction of coagulation parameters indicating absence of any inhibitors. Coagulation factor assay panel for intrinsic pathway showed severe deficiency of factor XII being 3% only (normal 70-15%). No other coagulation abnormality was detected.

**Final diagnosis:** sub-massive pulmonary embolism, hypoxemic respiratory failure associated with factor XII (Hageman Factor) deficiency.

### Management

Admitted to adult intensive care unit (AICU) for close monitoring: 1) Oxygen via face mask 6 - 8litres/min, targeting oxygen saturation above 92%; 2) Anticoagulation with injection clexane 60mg SC Q12Hrs 5 days, then after she was reported factor XII deficient, was started on oral tablet warfarin with therapeutic INR target ranged 2-3. Once INR value above 2 was achieved on warfarin, injection clexane was discontinued; 3) Intravenous fluids normal saline 50ml/hr; 4) Normal diet; 5) Tablet Paracetamol 1gm P.O Q8Hrs.

### Course in the hospital

She remained hemodynamically stable, and responded well to management, chest pain subsided within 24 hours of admission, gradually her supplementary oxygen requirement declined and after one week of treatment she was able to maintain acceptable oxygen saturation above 90% on spontaneous breathing at room air. Her family was counseled for screening of other family members for factor XII deficiency. After 9 days in AICU and requiring no more oxygen was shifted in general ward. Her laboratory work up was completed. Mixing studies showed no inhibitors while coagulation factor assay reported having factor XII deficiency associated with isolated raised activated partial thromboplastin time (aPPT). Subsequently after 15 days of hospitalization she was discharged in stable condition having O2 saturation of 98% at room air having no complaints. She was advised to take plenty of oral fluids as general advice and could continue lactating her baby. Out-patient follow up appointment given for anticoagulant clinic after 2 weeks with INR results.

## Discussion

In the intrinsic coagulation cascade, factor XII acts as an initiating step orchestrating enzymatic conversion of factor XI to activated factor Xia [7, 8]. Although majority is autosomal recessive as a result of spontaneous mutation, but autosomal dominant inheritance is also reported in the literature. This initial step is followed by thrombin stabilization, release of bradykinin and local inflammatory reaction [9, 10]. Causes of isolated elevated aPPT (activated partial thromboplastin time) include use of unfractionated-heparin, antiphospholipid syndrome, Von Willebrand varients, and deficiency of IX, XI, XII factors [11]. A mixing study and coagulation factor assay is contemplated to confirm the diagnosis. In vitro prolongation of aPPT may not represent increased risk of coagulopathy in vivo, because it is masked by other coagulation factors. Immunoassay is used directly to estimate the levels of factor XII [12]. No other coagulopathy was reported in this patient. The subject patient did not predate any symptoms related to factor XII deficiency, until her presentation with sudden chest pain and breathlessness due to sub-massive pulmonary embolism.

### Classification of pulmonary embolism

1) **Mild**, patients are hemodynamically stable and has normal echocardiograph. 2) **Sub-massive**, patients has relative hemodynamical stability (systolic BP is above 90mmHg) but are severely breathless, besides characteristic ECG changes may have abnormal cardiac enzyme rise, has acute dilatation of right ventricle with acute pulmonary hypertension and interventricular septal deviation on echocardiography. Our patient was in this category having all these features mentioned above and managed with anticoagulants. 3) **Massive**, patients are hemodynamically unstable (systolic BP < 90/40 for 15minutes or more), usually are managed with thrombolytic therapy if no contraindications.

Whereas both arterial as well as venous thromboembolic complications has been reported, though rarely, some authors have reported increased risk of bleeding [13]. On the other hand clinical use of heparins may catalyze activation of factor XII, thus prolonging aPPT. A prolonged aPPT in vitro has no relevance to clinically observed in vivo thromboembolic complications. According to the waterfall cascade hypothesis of coagulation, activation of intrinsic pathways is dependent on the presence of factor XII [14]. Paradoxically deficiency of factor XII leads to higher propensity for pro-coagulation rather than manifesting as coagulopathy. This paradoxical manifestations leads to increased number of acute events like myocardial infarction, miscarriages, new stroke or extension of strokes and pulmonary embolism in apparently healthy young patients as of our patient. A bleeding diathesis in the form of chronic subdural hematoma is suggested due to increased vascular permeability rather than coagulopathy [15]. An acquired factor XII deficiency has been reported as well, in patients having liver transplants [16].

Our patient did not report any pre-existing symptoms or history of any coagulopathy or acute event in the past. She was ambulant and asymptomatic being hospitalized and discharged, 3 weeks ago after an uneventful caesarian section as a primigravida. Diagnosed having sub-massive pulmonary embolism and respiratory failure associated with factor XII deficiency presentation with sudden breathlessness and chest pain. She was initially treated with low molecular weight heparin and subsequently replaced with oral warfarin.

## Conclusion

An isolated prolonged aPTT in a patient having thromboembolic event, is a pointer to factor XII deficiency as a rare harbinger of life threatening complications as in our patient. Due to the rarity of factor XII deficiency, and variation in its clinical presentations, other possible causes under the clinical circumstances (provocating and non-provocating) must be ruled out before definitely associating them with inherited coagulopathy. Transmission of autosomal recessive disorders, which put the pregnancy at risk, could be prevented by avoiding consanguineous marriages, necessitating counseling and education. Question remains whether such patients be offered extended anticoagulation, and if yes where is the place for new (non-anti-vit K dependent factors) anticoagulants if at all.

## Competing interests

The authors declare no competing interests.

## References

[1] Colman RW (2006). Are hemostasis and thrombosis two sides of the same coin?. J Exp Med.

[2] Schmaier AH (2008). The elusive physiologic role of Factor XII. J Clin Invest.

[3] Cichon S, Martin L, Hennies HC, Müller F, Van Driessche K, Karpushova A (2006). Increased activity of coagulation factor XII (Hageman factor) causes hereditary angioedema Type II. Am J Hum Genet.

[4] Riley RS Factor XII Deficiency. Pathology, Virginia Common Wealth University April 2005.

[5] Renne T, Gailani D (2007). Role of factor XII in hemostasis and thrombosis: clinical implications. Expert Rev Cardiovasc Ther.

[6] Factor XII deficiency National Organization for Rare Disorders (NORD.

[7] Pauer HU, Burfind P, Kostering H, Emons G, Hinney B (2003). Factor XII deficiency is strongly associated with primary recurrent abortions. Fertility and Sterility.

[8] Zeerleder S, Schooesser M, Redondo M, Wuillemin WA, Engel W, Furlan M (1999). Reevaluation of the incidence of thromboembolic complications in congenital factor XII deficiency a study on 73 subjects from 14 Swiss families. Thrombosis and Haemostais.

[9] Gailani D, Renne T (2007). Intrinsic pathway of coagulation and arterial thrombosis. Arteroscar Thromb Vasc Biol.

[10] Bork K (2006). Hereitary angioedema with normal C1 inhibitor activity including hereditary angioedema with coagulation factor XII gene mutations. Immunol Allergy Clin North Am.

[11] Gailani D, Broze GJ (1991). Factor XI activation in a revised model of blood coagulation. Science.

[12] Girolami A, Ruzzon E, Lombardi AM, Carbrio L, Randi ML (2004). Thrombosis free surgical procedures in severe (Homozygote) factor XII deficiency. Clin Appl Thromb Hemost.

[13] Joseph K, Tuscano TB, Kaplan AP (2008). Studies of the mechanisms of bradykinins generation in hereditary angioedema plasma. Ann Allergy Asthma Immunol.

[14] Pham M, Stoll G, Nieswandt B, Bendszus M, Kleinschnitz C (2012). Blood coagulation factor XII- a neglected player in stroke pathophysiology. J Mol Med (Berl).

[15] Colman RW (2006). Are hemostasis and thrombosis two sides of the same coin?. J Exp Med.

[16] Feng S, Goodrich NP, Bragg-Gresham JL, Dykstra DM, Punch JD, DebRoy MA (2006). Characteristics associated with liver graft failure: the concept of a donor risk index. Am J Transplant.

